# Clinical outcomes of proximal femoral reconstruction technique combined with THA in the treatment of high dislocation secondary to septic arthritis: a retrospective single-center study

**DOI:** 10.1186/s12891-023-06818-8

**Published:** 2023-09-14

**Authors:** Qingshan Xu, Qijin Wang, Jianfu Zhu, Jianguo Lin, Zhenbao Lu, Tihui Wang, Xu Wang, Qiujin Xia

**Affiliations:** Department of Orthopedics, Affiliated Mindong Hospital of Fujian Medical University, Ningde, 355000 Fujian China

**Keywords:** Septic arthritis, Sequelae, Total hip arthroplasty, Proximal femur reconstruction

## Abstract

**Purpose:**

The aim of this retrospective study was to examine the clinical outcomes and complications of proximal femur reconstruction (PFR) combined with total hip arthroplasty (THA) in patients with high hip dislocation secondary to septic arthritis (SA).

**Methods:**

Between September 2016 to September 2021, we performed a series of 15 consecutive PFR combined with THA on patients with high dislocation of the hip secondary to SA, of these,12 hips were reviewed retrospectively, with a mean follow-up of 2.5 years (range, 1.5-6 years). The mean age of the patients at the time of surgery was 52 years (range, 40–70 years).

**Results:**

All patients were followed up. At 1-year postoperative follow-up, the median HHS increased from 32.50 preoperatively to 79.50 postoperatively. The median VAS decreased from 7 before surgery to 2 at 1 year after surgery. The median LLD reduced from 45 mm preoperatively to 8 mm at 1 year after surgery. The mean operative time 125 ± 15 min (range 103-195 min). Mean estimated blood loss was500 ± 105ml (range 450–870 ml). Mean hospital days 9.5 days (range 6–15 days). Two patients developed nerve injuries that improved after nutritional nerve treatment. One patient had recurrent postoperative dislocation and underwent reoperation, with no recurrence dislocation during the follow-up. There were no cases of prosthesis loosening during the follow-up period. One patient developed acute postoperative periprosthetic joint infection (PJI) that was treated with Debridement, Antibiotics and Implant Retention (DAIR) plus anti-infective therapy, with no recurrence during 2 years of follow-up.

**Conclusion:**

This study indicates PFR combined with THA shows promise as a technique to manage high hip dislocation secondary to SA, improving early outcomes related to pain, function, and limb length discrepancy.

## Introduction

Septic arthritis (SA) of the hip is a common pediatric disease in less developed countries [1]. High hip dislocation is one of a serious potential sequelae of SA, which can lead to impaired ambulation and reduced quality of life for patients. This is due to abnormal hip anatomy, scarring andcontractures of surrounding soft tissues, severe lower limb length discrepancy ( LLD), and pelvic tilt [2, 3].

Total hip arthroplasty (THA) is an effective treatment for high hip dislocation secondary to SA [4]. The procedure should account for acetabular and femoral dysplasia, restoration of the hip’s anatomical center of rotation, and leg length equalization. Thus, accurate placement of the acetabular component in the true acetabulum and restoring the hip’s native center of rotation during THA is critical for long-term prosthetic survival [1, 5]. However, this can be technically demanding. Previous studies have reported that shortening subtrochanteric osteotomy (SSO) can effectively restore the hip joint’s center of rotation and hip function [6, 7]. However, SSO remains challenging in patients with chronic high hip dislocation. Extensive contracture and scarring of the severely dysplastic hip and surrounding soft tissues leads to poor surgical visualization. This makes it difficult to identify anatomy and original hip center location [8].

Proximal femoral reconstruction(PFR) [9] involves performing an oblique osteotomy using a long, angled oscillating saw. This osteotomy is directed downward on the medial aspect of the greater trochanter, forming an 8–14 cm bone block consisting of the gluteus medius, gluteus minimus, and gluteus lateralis muscles. PFR can fully expose both the acetabular and femoral regions, reducing surgical difficulty and operative time. It also enables sliding the bone block up and down to position the greater trochanter fragment in moderate tension, reconstructing the proximal femur anatomy. This may improve abductor function and adjust leg length discrepancy, facilitating restoration of hip joint function. Prior studies have reported satisfactory early postoperative outcomes using PFR combined with THA for Crowe type IV developmental dysplasia of the hip (DDH) [9].

Currently, there have been limited reports on the use of PFR combined with THA for treating high hip dislocation secondary to SA. The present study retrospectively analyzed short- and medium-term outcomes in patients treated with PFR combined with THA for high hip dislocation secondary to SA at our institution. The aim was to investigate the efficacy and safety of this combined procedural approach.

## Materials and methods

### Patient selection

This retrospective study was approved by the institutional ethics committee at our institution, and informed consent was obtained from all patients (2,023,033,001 K). Between September 2016 and October 2021, a total of 15 patients with high hip dislocation secondary to SA underwent PFR combined with THA at our institution. The inclusion and exclusion criteria were as follows. Inclusion criteria were: patients with osteoarthritis of the high dislocated hip requiring surgical treatment, with a clear previous history of acute septic disease of the hip. Exclusion criteria were: patients with DDH or other etiologies causing high dislocation osteoarthritis. SA was defined as a prior history of the hip joint exhibiting redness, swelling, heat, pain, discharge, sinus tract formation, or positive joint aspirate cultures.

### Preoperative evaluation

All patients underwent preoperative radiographic imaging including hip X-rays and CT scans with 3D reconstruction. These studies helped determine the degree of acetabular and femoral deformity, femoral head dislocation, and proximal femoral medullary cavity anatomy. This allowed assessment of bone stock and guided acetabular reconstruction planning and appropriate prosthesis selection. Preoperative lab work included white blood cell count, C-reactive protein level, and erythrocyte sedimentation rate.

### Intraoperative testing

Prior to arthrotomy, synovial fluid was aspirated using sterile technique for cell count and culture. After opening the joint, deep tissue samples were collected for histopathologic examination and microbiologic culture. Active infection was diagnosed if at least 2 of the following were present: gross purulence, > 5 PMNs per high power field on pathology, and positive cultures from at least 2 deep tissue samples.

### Perioperative care

Oral celecoxib and dextrozopiclone were given the night before surgery. Intravenous cefuroxime and tranexamic acid were given 30 min preoperatively. Redosing of antibiotics occurred for operations exceeding 3 h or blood loss greater than 800mL. Postoperative antibiotic prophylaxis was continued for 24 h. Urinary catheterization was utilized perioperatively until postoperative day 1. Closed suction drainage was removed when output was < 50 mL per day. Intraoperatively, vancomycin and meropenem powder was applied to the prosthetic components before implantation as infection prophylaxis.

### Surgical technique

After successful anesthesia induction, the patient was positioned supine. The surgical site was prepped and draped in a sterile fashion. A small incision was made over the adductor muscles, which were then released to facilitate hip abduction. The incision was closed with sutures. With the patient in the lateral decubitus position, a 15 cm posterior lateral incision was made over the hip. The incision went through the skin, subcutaneous tissue and deep fascia, along the posterior border of the gluteus medius. The external rotators were exposed and a longitudinal incision was made, releasing them posteriorly to reveal the hip joint capsule. A T-shaped capsulotomy was performed to access the femoral neck and head. Retractors were placed to protect the gluteus medius. An oblique osteotomy was performed on the proximal femur, starting 8–14 cm above the lesser trochanter and directed laterally. Scar tissue was released and care taken to preserve 1 cm of the calcar femorale. If needed, a shortening subtrochanteric osteotomy was done. The femoral head was then removed. Under C-arm guidance, the true acetabulum was identified. With the leg internally rotated, reaming was performed with care to minimize abrasion of viable bone. The transverse ligament were identified. The appropriate reamer size was selected to remove articular cartilage down to bleeding subchondral bone based on the amount of native acetabular bone stock.

After trialing acetabular components, an appropriate porous coated hemispherical acetabular shell was selected. The acetabulum was sequentially reamed to accept the prosthesis. Shell position and depth were verified with measurements. Two fixation screws were placed and the corresponding polyethylene liner was impacted into place.

With the hip flexed and internally rotated, the osteotomized distal femur was temporarily fixed with wires. The femoral canal was opened with sequential reamers to the appropriate size based on preoperative templating. The femoral canal was thoroughly irrigated. The femoral stem was inserted with porous coating directed laterally and the modular femoral head was impacted onto the trunnion. Traction was released and the hip trialed through a range of motion to verify stability. The greater trochanteric osteotomy was repaired using wires to hold the proximal fragment to the lateral femoral cortex. The hip joint capsule and external rotators were repaired to provide soft tissue stability. The fascia, subcutaneous tissue and skin were closed in layered fashion. Stability of the prosthetic joint was verified and the wounds were dressed in sterile fashion.

### Postoperative functional exercises

All patients are encouraged to engage in early postoperative activity and limb exercises immediately after the surgery. Partial weight-bearing walking is permitted for approximately 2 weeks, followed by a gradual progression to full weight-bearing walking between 4 and 6 weeks post-surgery, based on the stability of the femoral shaft and the healing condition of the surgical site.

### Outcome measures

Clinical assessment utilized Harris Hip Score (HHS) [10], and the visual analog scale (VAS) for pain [11]. Outcomes recorded included operative time, blood loss, length of hospital stay, nerve injury incidence, hip dislocation rate, prosthesis loosening, infection rate.

### Statistical analysis

Statistical analyses were performed using SPSS 22.0 (SPSS Inc., Chicago, IL, USA). Categorical variances were expressed as frequencies, while continuous variables were expressed as means and ranges. Chi-squared test were utilized to compare preoperative and postoperative categorical data. Paired t-test were used to compare preoperative and postoperative continuous data. with P＜0.05 were considered statistically significant.

## Results

### Demographic characteristics

According to the inclusion and exclusion criteria, 15 patients were initially recruited. One patient was lost to follow-up, one declined participation, and one had incomplete data. Thus, 12 cases were included in the analysis. There were 7 males and 5 females; all patients had a positive Trendelenburg sign. Intraoperative cultures of surgical specimens were negative for all patients. Non-cemented, biologically fixed prostheses were utilized for all cases, include: Summit stem (DePuy Synthes, Warsaw, IN), Pinnacle Gription acetabular cup with Altrx liner (DePuy Synthes, Warsaw, IN) and Ceramic femoral head (CeramTec, Plochingen, Germany). Twelve patients were followed up after surgery with a mean follow-up time of 2.5 ± 0.72 years (range, 1.5-6 years). Patient demographics are presented in Table 1.

**Table 1:** Patient demographic characteristics

Demographic characteristics	Values
Mean age (years)	52 ± 13
Sex	
Male	7
Female	5
BMI(kg/m2)	22.99 ± 2.60
Duration of infection quiescence (years)	24 ± 11.5
Crowe type (dislocation of the hip)	
III	4
IV	8
Mean follow-up time ( years)	2.5 ± 0.72(range,1.5-6)
comorbidities	
Smoking (Yes/No)	2/10
Drinking alcohol (Yes/No)	2/10
High blood pressure (Yes/No)	5/7
Diabetes (Yes/No)	4/8

### Clinical outcomes

The mean operative time was 125 ± 15 min (range 103–195 min). The mean estimated blood loss was 500 ± 105 mL (range 450–870 mL). Three patients required transfusion of 2 units of leukocyte-depleted erythrocytes. The mean hospital stay was 9.5 days (range 6–15 days). At 1-year postoperative follow-up, the median HHS increased from 32.50 preoperatively to 79.50 postoperatively. The median VAS decreased from 7 before surgery to 2 at 1 year after surgery. The median LLD reduced from 45 mm preoperatively to 8 mm at 1 year after surgery. In summary, the medians of HHS, VAS, and LLD all showed improvement at 1-year follow-up compared to preoperative values. (Table 2). Five patients achieved full weight-bearing at 6 weeks postoperative, while seven patients achieved full weight-bearing at 3 months postoperative. PFR osteotomy site healing occurred within 3–6 months.

**Table 2:** Comparison of HHS and VAS scores and LLD of patients before and 1 year after surgery (${\bar x }$± s)

Variable	Preoperative	1 year after surgery	t	p
HHS	32.25 ± 4.20	79.17 ± 7.43	-14.407	0.001
VAS	7.00 ± 1.04	2.00 ± 0.67	13.428	0.001
LLD(mm)	46.25 ± 5.60	7.50 ± 1.38	28.26	0.001

HHS: Hip Harris Score; VAS: visual analogue scoring; LLD: lower limb length difference

### Complications

One patient developed femoral nerve injury, which improved symptomatically after 6 months of nutritional nerve therapy. Another patient experienced sciatic nerve palsy, which also improved symptomatically after 7 months of nerve nutrition treatment. One patient had recurrent dislocation within 1 month postoperatively, which was found to be obstructed by a bone fragment. After removal of the obstructing fragment, no further dislocations occurred over 3 years of follow-up. There was no evidence of prosthetic loosening during follow-up. One patient developed incisional wound drainage at 2 weeks, with joint aspirate cultures positive for periprosthetic joint infection (PJI). This was treated with debridement, antibiotics, and implant retention (DAIR) plus antibiotic therapy, with no recurrence at 2 years follow-up.

### Typical case

**Fig. 1:**
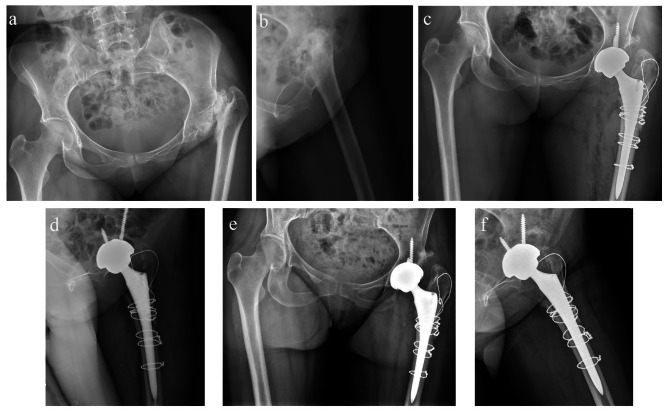
**a**, **b** preoperative anteroposterior pelvic radiographs; **c**, **d** Hip X-ray after left proximal femur reconstruction + THA; **e**, **f** X-ray reexamination 6 months after surgery indicated that the proximal femur had osseous union

**Fig. 2:**
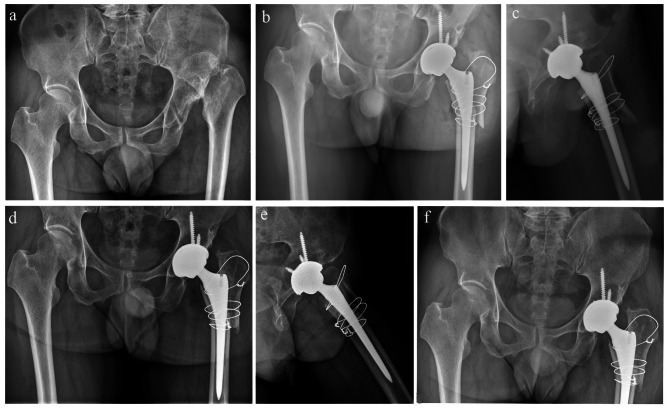
**a** preoperative anteroposterior radiography of the pelvis; **b**, **c** X-ray after reconstruction of proximal left femur + THA; **d**, **e** X-ray reexamination 6 months after surgery showed that the proximal femur reconstruction had basically healed. **f** at the 1-year postoperative follow-up, the prosthesis was in good position

The patient was a 62 years old female with left hip pain with claudication for 55 years. The orthopantomogram of the outpatient pelvis is shown below (Fig. 1a,b). At the age of 7, the left hip was red, swollen, hot, painful, and functionally restricted with sinus tract formation, and was treated with surgical debridement combined with antibiotics. The patient had left hip pain with limited movement, hip flexion inversion, limited abduction, limping, and left lower limb shortening of about 42 mm, which seriously affected the quality of life and required surgical treatment, and underwent left PFR combined with THA at our institution (Fig. 1c, d). At the 6-month postoperative follow-up, the prosthesis was in good position and the osteotomy site had healed (Fig. 1e, f). The other patient was 56 years old and had left hip pain with claudication for 45 years. The orthopantomogram of the outpatient pelvis is shown in the figure below (Fig. 2a). The patient had left hip pain with obvious movement, hip flexion and adduction, limited abduction, limping, and shortening of the left lower limb by about 49 mm, which seriously affected the quality of life, and she came to our institution for left proximal femoral reconstruction combined with THA (Fig. 2b, c), after which the lower limb was basically equal in length. At the 6-month postoperative follow-up, the PFA reconstruction site had achieved bony union (Fig. 2d, e). At the 1-year postoperative follow-up, the prosthesis was in good position. (Fig. 2f).

## Discussion

### Surgical approach and timing for high hip dislocation secondary to SA

Treatment options for high hip dislocation secondary to septic arthritis include: (1) THA, which presents surgical exposure challenges, inability to place the acetabular component in the true socket, and postoperative limb length inequality; (2) SSO combined with THA, which has been reported to effectively reconstruct the hip center of rotation and restore abductor function and limb length equality [12], however acetabular exposure remains difficult and osteotomy nonunion can occur [13]; (3) PFR combined with THA, which utilizes an oblique proximal femur osteotomy to mobilize the abductors and greater trochanter posteriorly and superiorly, improving acetabular exposure [9].

In the present study, all patients were treated with PFR combined with THA. The medians of HHS, VAS, and LLD at 1 year postoperatively showed improvements compared to preoperative values. One patient (4.1%) experienced transient sciatic nerve palsy and one had femoral nerve palsy, both recovering by 6 and 8 months postoperatively, respectively, after nutritional nerve therapy without residual deficits. These findings are comparable to a prior study by Yue Luo et al. [1], who attributed the potential causes for transient nerve injury to substantial limb lengthening, surgical inexperience, and prolonged operative times.

Some studies have suggested that lower limb lengthening exceeding 3.5 cm increases the risk of nerve injury [1, 14], while others propose nerve injury may occur with lengthening beyond 4 cm [15, 16]. In the present study, the mean limb lengthening was approximately 3.9 cm, with a maximum of 4.6 cm in one patient who did not experience nerve injury. The other two patients with nerve injuries had 4.1 cm and 3.9 cm of lengthening, respectively. We hypothesize the severe soft tissue contractures in these cases necessitated extensive releases, hampering easy repositioning of the hip joint and placing the nerves under excessive traction during manipulation. Therefore, careful repositioning technique is critical to avoid nerve injury in patients with severe contractures, even after adequate surgical releases, given the persistent difficulty with hip reduction.

There is no established definitive timeframe for performing arthroplasty after septic arthritis. Most experts recommend allowing over 10 years after the infection before attempting total hip arthroplasty to minimize the risk of recurrent infection [17–19]. Additionally, comprehensive histological and microbiological sampling is necessary preoperatively to maximize detection of any residual bacteria. Preoperative assessment should include inflammatory markers like complete blood count, erythrocyte sedimentation rate (ESR), and C-reactive protein (CRP), along with intraoperative sampling of tissues for bacterial cultures and frozen section analysis.

Intraoperative frozen section analysis showing over 5 neutrophils per high-powered field indicates a high index of suspicion for active infection. In this study, all patients had normal preoperative inflammatory markers, negative intraoperative cultures, and frozen sections with less than 5 neutrophils per high power field. The mean interval between prior infection and arthroplasty was 24 ± 11.5 years (range 16–60 years). Hematologic parameters, frozen sections, synovial fluid, and surgical specimen cultures showed no evidence of residual bacterial infection perioperatively. However, one patient developed incisional wound drainage with marked erythema at 2 weeks postoperatively; arthrocentesis grew Staphylococcus epidermidis, which was treated with debridement, antibiotics, and implant retention plus antibiotic therapy without recurrence at 2-year follow-up.

### PFR allows better visualization of the lateral acetabulum

For patients with high hip dislocation secondary to severe acetabular dysplasia, it is crucial to obtain a clear intraoperative view of the acetabular side of the hip, which can simplify the surgery [20]. The SSO often does not provide a clear surgical view, making the surgery difficult. In contrast, peri-femoral osteotomy is a long oblique osteotomy with the proximal femur angled outward and downward, and this osteotomy can pull the intact bone block of the gluteus medius, gluteus minimus and vastus lateralis outward and upward as a whole, which can fully expose the acetabulum and simplify the surgery.

There is debate regarding whether the acetabular prosthesis should be positioned at the level of the true acetabulum or the false acetabulum in these patients. Most scholars advocate reconstructing the acetabulum at the level of the native acetabulum or slightly proximal to restore normal anatomical relationships and hip biomechanics [21, 22]. However, some argue that patients with longstanding dislocation have adapted to non-physiological hip motion patterns, thus eliminating the need to precisely reconstruct the anatomical acetabulum. These scholars suggest it is safe and effective to implant the prosthetic acetabulum slightly superiorly (less than 20 mm) within the native acetabulum [23].

However, excessive superior positioning of the acetabular prosthesis may impair function of the hip abductors, negatively impacting ambulation. It can also lead to abnormal forces on the prosthesis, resulting in early component loosening. Obtaining adequate bone coverage of the acetabular component is important. There is no consensus on the degree of host bone coverage needed for the acetabular prosthesis. Mulroy and Harris [24] found that less than 40% host bone coverage is associated with higher failure rates. They recommended at least 70% coverage by host bone to maintain stability and allow adequate ingrowth.

Placing the acetabular prosthesis as centrally as possible in the acetabulum and obtaining adequate bone coverage ensures a longer duration of use of the acetabular prosthesis. In this study, all patients underwent rigorous preoperative planning, adequate assessment of periacetabular bone volume, and intraoperative C-arm machine fluoroscopy. 66.7% (8/12) of patients were placed in the true socket position. 3 patients were appropriately displaced upward in the acetabulum with a smaller acetabular cup due to insufficient bone volume in the true socket. The other 1 case was appropriately upwardly displaced in an internally displaced acetabulum.

It is ensured that the host bone covers over 70% of the acetabular prosthesis surface. We believe reconstruction should be performed at the level of the true acetabulum when sufficient bone stock exists; if bone stock is insufficient in the true acetabulum, controlled superior and medial displacement of the prosthesis is reasonable, and further studies are warranted to determine the degree of upward displacement that does not impair hip joint function. Additionally, when reaming the acetabulum, the initial reamer size is the smallest possible followed by sequential incremental reaming. Repeated assessment of the periprosthetic bone stock is necessary, as the anterior acetabular wall is often thin while the posterior wall is thick. Therefore, the reaming process is directed posteriorly to avoid penetrating the anterior cortex. Final backside reaming provides optimal prosthetic fixation by compacting the bone bed.

In this study, one patient experienced three dislocations within 2 months after the initial surgery. Reoperation revealed bony redundancy obstructing the prosthetic joint superiorly and posteriorly. No further dislocation occurred for 2 years after surgical excision of the bony blockage. We analyzed possible causes in this case of hip arthroplasty for septic sequelae. The initial joint replacement was performed when the periprosthetic soft tissues were severely contracted and stiff, placing the hip under high tension with restricted mobility. Postoperatively, as joint motion exercises restored elasticity of the soft tissues, mobility increased, allowing dislocation in the presence of bony obstruction. Therefore, we recommend maintaining appropriate intraoperative tension in similar cases and thoroughly inspecting for potential impinging bony landmarks around the prosthetic acetabulum, which should be removed to prevent instability.

### PFR allows better visualization of the lateral femur

In patients with high hip dislocation secondary to SA, improved surgical exposure of the femoral side facilitates implantation of the femoral prosthesis and adjustment of the optimal femoral anteversion angle. However, severe anatomical deformities often exist, including soft tissue contractures from prior infections and repeated operations [20], aberrant locations of the peripheral nerves and blood vessels, and narrowing or occlusion of the proximal femoral canal. These factors can impede reaming of the femoral cavity and insertion of the prosthesis.

Therefore, reduction of the femoral head into the true acetabulum without neurovascular injury is challenging. The conventional transverse subtrochanteric osteotomy has limited bone contact and inherent rotational stability, risking nonunion [13]. To increase osseous apposition and avoid rotational instability after transverse osteotomy, various modified subtrochanteric osteotomies have been proposed, including oblique [25], Z-shaped [26], and V-shaped cuts [27].

However, modified subtrochanteric osteotomies have not significantly improved bone healing [28]. The PFR involves an oblique cut of 8–14 cm length with distal angulation, elevating the proximal femur and attached muscles en bloc to expose the lateral femur. This not only facilitates canal preparation and appropriate femoral version, but also allows adjustment of the osteotomy fragment to restore abductor tension. Additionally, a large surface area of bone contact can be obtained for reconstruction. Morselized autograft or resected femoral head graft implanted at the osteotomy site and securely fixed with cerclage wiring can accelerate healing and enhance rotational stability. In this study, osseous union was achieved at 3–6 months after peri-femoral reconstruction in all cases.

It should be noted that patients with high hip dislocation secondary to SA have poor cortical bone quality, thus femoral canal preparation and prosthetic insertion risks iatrogenic fracture. Therefore, prophylactic cerclage wiring of the proximal femur before reaming and gentle maneuvering are imperative. Notably, most high hip dislocation patients treated with SSO require expensive S-ROM components. In contrast, proximal femoral reconstruction can be performed using standard implants, reducing costs and potentially improving patient satisfaction.

Moreover, to address limb length inequality, preoperative full-length radiographs of both lower extremities should be obtained for surgical planning. Intraoperatively, femoral prosthetic position is determined in reference to the lesser trochanter, followed by sufficient soft tissue releases. Most patients with limited abduction require a femoral adductor tenotomy. During reduction, an assistant stabilizes the pelvis from the anterior superior iliac crest while the surgeon applies traction to the limb. Another assistant aids the maneuver by manipulating the femoral resurfacing component.Forceful reduction maneuvers should be avoided. If repeated closed reductions fail, the areas of tension can be marked and released under traction to the lower limb. If reduction remains impossible after releases, downsizing the femoral component or utilizing a reduced femoral head size are options, albeit with increased risk of leg length inequality and instability postoperatively. Thus, rotator and posterior soft tissue repair should be emphasized intraoperatively. In this study, one patient underwent successful relocation after exchanging to a shorter femoral head following repeated failed closed reductions and soft tissue releases. At 3 years postoperatively, no recurrence of dislocation had occurred.

## Limitations

The main limitations of this study are its retrospective design, small number size, lack of control group, and short follow-up period. However, to our knowledge, this is the first short- to medium-term report of proximal femoral reconstruction combined with THA in patients with high hip dislocation secondary to SA. Future studies should include more cases and controls.

## Conclusion

This retrospective study provides preliminary evidence suggesting that the combined approach of PFR and THA may represent a potential therapeutic option for high hip dislocation secondary to SA, improving early outcomes related to pain, function, and limb length discrepancy.

## Data Availability

The datasets generated during and/or analyzed during the current study are not publicly available, but are available from the corresponding author on reasonable request.
